# New *Halonotius* Species Provide Genomics-Based Insights Into Cobalamin Synthesis in Haloarchaea

**DOI:** 10.3389/fmicb.2019.01928

**Published:** 2019-08-27

**Authors:** Ana Durán-Viseras, Adrian-Stefan Andrei, Rohit Ghai, Cristina Sánchez-Porro, Antonio Ventosa

**Affiliations:** ^1^Department of Microbiology and Parasitology, Faculty of Pharmacy, University of Seville, Seville, Spain; ^2^Department of Aquatic Microbial Ecology, Institute of Hydrobiology, Biology Centre of the Academy of Sciences of the Czech Republic, České Budějovice, Czechia

**Keywords:** *Halonotius*, haloarchaea, comparative genomic analysis, hypersaline environment, *Halonotius terrestris* sp. nov., *Halonotius roseus* sp. nov.

## Abstract

Hypersaline aquatic and terrestrial ecosystems display a cosmopolitan distribution. These environments teem with microbes and harbor a plethora of prokaryotic lineages that evaded ecological characterization due to the prior inability to cultivate them or to access their genomic information. In order to close the current knowledge gap, we performed two sampling and isolation campaigns in the saline soils of the Odiel Saltmarshes and the salterns of Isla Cristina (Huelva, Spain). From the isolated haloarchaeal strains subjected to high-throughput phylogenetic screening, two were chosen (F15B^T^ and F9-27^T^) for physiological and genomic characterization due of their relatedness to the genus *Halonotius*. Comparative genomic analyses were carried out between the isolated strains and the genomes of previously described species *Halonotius pteroides* CECT 7525^T^, *Halonotius aquaticus* F13-13^T^ and environmentaly recovered metagenome-assembled representatives of the genus *Halonotius*. The topology of the phylogenomic tree showed agreement with the phylogenetic ones based on 16S rRNA and *rpoB′* genes, and together with average amino acid and nucleotide identities suggested the two strains as novel species within the genus. We propose the names *Halonotius terrestris* sp. nov. (type strain F15B^T^ = CECT 9688^T^ = CCM 8954^T^) and *Halonotius roseus* sp. nov. (type strain F9-27^T^ = CECT 9745^T^ = CCM 8956^T^) for these strains. Comparative genomic analyses within the genus highlighted a typical *salt-in* signature, characterized by acidic proteomes with low isoelectric points, and indicated heterotrophic aerobic lifestyles. Genome-scale metabolic reconstructions revealed that the newly proposed species encode all the necessary enzymatic reactions involved in cobalamin (vitamin B_12_) biosynthesis. Based on the worldwide distribution of the genus and its abundance in hypersaline habitats we postulate that its members perform a critical function by being able to provide “expensive” commodities (i.e., vitamin B_12_) to the halophilic microbial communities at large.

## Introduction

Haloarchaea are extremely halophilic archaea belonging to the phylum *Euryarchaeota*. They constitute a diverse group of archaea that are widely distributed in different extreme habitats with high salt concentrations, from aquatic systems (saline lakes, salterns) to saline soils, salt mines and salted foods (Ventosa, 2006). Haloarchaea are placed in the class *Halobacteria*, which comprises three orders: *Halobacteriales*, *Haloferacales* and *Natrialbales*, encompassing five families and more than 50 genera (Amoozegar et al., 2017). Despite their ecological role (in hypersaline habitats they constitute a dominant population) (Ghai et al., 2011; Fernández et al., 2014a, b; Ventosa et al., 2015; Vera-Gargallo et al., 2019) and their biotechnological potential (Amoozegar et al., 2017), little information is available on the genomic architecture and metabolic potential of cultured haloarchaea representatives (Bolhuis et al., 2006; Pfeiffer et al., 2008). The genus *Halonotius* belongs to the class *Halobacteria*, order *Haloferacales*, family *Halorubraceae* (Amoozegar et al., 2017) and currently it includes two species: the type species, *Halonotius pteroides*, isolated from a crystallizer of an Australian saltern, which represented up to 16% of the prokaryotic population of this habitat (Burns et al., 2004, 2010), and *Halonotius aquaticus*, recently isolated from a marine saltern in Spain (Durán-Viseras et al., 2019). Although the genus comprises only two described species, it has been previously reported in several other habitats, such as saline lakes in Australia and China (Podell et al., 2014; Han et al., 2017), solar salterns in Turkey and Spain (Çınar and Mutlu, 2016; Durán-Viseras et al., 2019) and food-grade salt samples (Henriet et al., 2014), indicative of a world-wide environmental distribution. Moreover, three metagenome-assembled genomes previously recovered from Lake Tyrell (Australia), appeared to be phylogenetically close to *Halonotius* (Podell et al., 2013).

In this work, we have carried out a comparative genomic analysis of the genus *Halonotius*, for which we used the genomes of the two species belonging to the genus (i.e., *Halonotius pteroides* and *Halonotius aquaticus*), the ones from the two new strains that we isolated, and two metagenome-assembled genomes mentioned above. Our data indicate that *Halonotius* spp., like many other haloarchaea [e.g., *Haloquadratum* (Bolhuis et al., 2006), *Halobacterium* (Pfeiffer et al., 2008)], are photoheterotrophs with a typical aerobic electron transport chain and harbor a rhodopsin-related gene pool, specifically haloarchaeal proton-pumps and sensory rhodopsins. Remarkably, all analyzed *Halonotius* genomes were found to encode a complete cobalamin biosynthesis pathway. Moreover, our results show that this genus is abundant and diverse in hypersaline environments and support the description of two new species, for which we propose the designations *Halonotius roseus* sp. nov., and *Halonotius terrestris* sp. nov., respectively. Additionally, the detailed study of the polar lipids profiles of these strains justify an emendation of the genus *Halonotius*.

## Materials and Methods

### Strains Isolation

Two *Halonotius* strains were isolated during two sampling campaigns, conducted in September 2014 and June 2016, as previously described (Vera-Gargallo and Ventosa, 2018; Durán-Viseras et al., 2019). Strain F15B^T^ was isolated from a hypersaline soil (36.1 g/L salinity) of the Odiel Saltmarshes (Huelva, Spain, 37°22′N 6°98′W), while strain F9-27^T^ was isolated from a pond water sample (310 g/L salinity and pH 7.1) collected from the saltern of Isla Cristina (Huelva, Spain, 37°21′N 7°33′W). Samples were diluted, plated and incubated at 37°C for 8 weeks. Strains were isolated in R2A medium (Difco) supplemented with 25% (w/v) seawater salt solution prepared by dilution of SW 30% stock solution (Subow, 1931) which contained (g/L): NaCl, 195; MgCl_2_.6H_2_O, 32.5; MgSO_4_.7H_2_O, 50.8; CaCl_2_, 0.83; KCl, 5.0; NaHCO_3_, 0.17; NaBr, 0.58 and solidified with 2% (w/v) purified agar when necessary. The cultures were routinely grown in R2A 25% (w/v) medium adjusted to pH 7.5 and incubated at 37°C. They were maintained at −80°C in this medium containing 50% (v/v) glycerol.

*Halonotius pteroides* CECT 7525^T^, type species of the genus *Halonotius* and *Halonotius aquaticus* F13-13^T^ were also used in this study. These strains were also grown in R2A 25% (w/v) medium as described above.

### DNA Extraction, Purification and Sequencing

Genomic DNA was extracted and purified following the methodology previously described by Durán-Viseras et al. (2019). DNA quantification was determined using spectrophotometric (DeNovix DS-11 FX, DeNovix Technologies, Wilmington, DE, United States) and fluorometric (Qubit 3.0 Fluorometer, Thermofisher Scientific, United States) assays. DNA quality was checked by agarose gel (1%) electrophoresis.

The 16S rRNA and the *rpoB*′ genes were PCR-amplified, using the primer pairs ArchF/ArchR (DeLong, 1992; Arahal et al., 1996) and rpobF/rpobR (Fullmer et al., 2014), respectively, and sequenced by Sanger technology at a commercial company (Stabvida, Oeiras, Portugal). The shotgun sequencing of strains F15B^T^ and F9-27^T^ was also performed by the same commercial company (Stabvida, Oeiras, Portugal) on the Hiseq 4000 platform, using 150 bp paired-end sequencing. The genomes of the strains *Halonotius aquaticus* F13-13^T^ and *Halonotius pteroides* CECT 7525^T^ were recovered from a previous study (Durán-Viseras et al., 2019).

### Genome Assembly and Annotation

Preprocessing of raw Illumina reads of strains F15B^T^, F9-27^T^, *Halonotius aquaticus* F13-13^T^ and *Halonotius pteroides* CECT 7525^T^ was carried out by using a combination of software tools implemented in the BBMap project (Bushnell, 2016). Briefly, bbduk.sh was used to: (i) remove poor quality sequences from the obtained interleaved files (qtrim = rl trimq = 18), (ii) rename the reads, (iii) identify and remove phiX and p-Fosil2 controls (k = 21 ref = vectorfile ordered cardinality) and (iv) remove Illumina adapters (*k* = 21 ref = adapterfile ordered cardinality). bbmerge.sh was used for *de novo* identification of any other potential adapters. A total of, 5,402,062, 14,332,336, 13,117,040, and 14,471,142 reads were generated for the strains F15B^T^, F9-27^T^, *Halonotius aquaticus* F13-13^T^ and *Halonotius pteroides* CECT 7525^T^, respectively.

The quality-checked paired-end Illumina reads for the strains F15B^T^, F9-27^T^, *Halonotius aquaticus* F13-13^T^ and *Halonotius pteroides* CECT 7525^T^ were assembled using Spades (v3.12.0) (Bankevich et al., 2012) in 26, 4, 10, and 42 contigs, respectively. Assembly quality and genomic G + C content was checked using QUAST v2.3 (Gurevich et al., 2013). Genome completeness, contamination and strain heterogeneity were estimated using CheckM v1.0.5 (Parks et al., 2015). Genome sequences were annotated with Prokka (Seemann, 2014). BlastKOALA (Aziz et al., 2008) was used to assign KO identifiers (K numbers) to orthologous genes present in the genomes. Inferences of metabolic pathways were conducted by mapping to the KEGG pathways and KEGG modules the assigned KO numbers of each individual genome. Additionally, selected proteins were analyzed using jackhmmer (Finn et al., 2015), CDD (Marchler-Bauer et al., 2015), and Phobius (Kall et al., 2007).

### Phylogenetic Analyses

The 16S rRNA gene-based phylogenetic analyses were performed using the maximum-likelihood and maximum-parsimony algorithms integrated in the ARB software package (Ludwig et al., 2004). The 16S rRNA gene sequences alignment was confirmed and checked against both the primary and secondary structures of the 16S rRNA sequence using the alignment tool of the ARB software package. In order to evaluate the robustness of the obtained phylogenetic tree, a bootstrap analysis (1000 replications) was performed.

The *rpoB*′ gene-based phylogenetic analyses were performed by MEGA 6.0 software (Tamura et al., 2013) using the maximum-likelihood. In order to evaluate the robustness of the obtained phylogenetic tree, a bootstrap analysis (1000 replications) was performed.

For phylogenomic analyses a set of 257 conserved proteins, identified based on COG annotations (Tatusov et al., 2001), were individually aligned using PRANK (Löytynoja, 2014) (−protein + F), trimmed with BMGE (Criscuolo and Gribaldo, 2010) (−m BLOSUM30 –t AA –g 0.5) and concatenated. The obtained alignment (containing 40,476 positions) was used for maximum-likelihood phylogeny reconstruction using IQ-TREE (version 1.6.7) (Nguyen et al., 2015): amino-acid exchange rate matrix LG (Le and Gascuel, 2008), empirical base frequencies and discrete Gamma rate heterogeneity (as selected by ModelFinder as the best-fit substitution model; Kalyaanamoorthy et al., 2017). The tree topology was tested using SH-Alrt and ultrafast bootstrap approximation (1000 replications).

**FIGURE 1:**
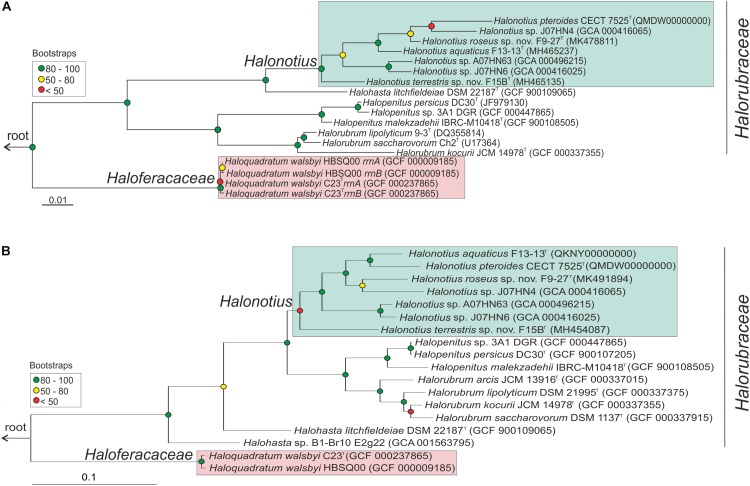
*Halonotius* phylogenetic analysis. **(A)** Maximum-likelihood phylogenetic tree based on the 16S rRNA gene sequences. Bar, 0.01 substitutions per nucleotide position. **(B)** Maximum-likelihood phylogenetic tree based on amino acid sequences of the *rpoB*′ genes. Bar, 0.1 substitution per nucleotide position. Sequence accession numbers are shown in parentheses. Red circles highlight Bootstrap/UFBootstrap values lower than 50, the yellow ones between 50 and 80 and green color is used to depict values higher than 80. The trees were rooted using the DPANN representatives *Candidatus* Nanosalinarum J07AB56 and *Candidatus* Nanosalina J07AB43.

**FIGURE 2:**
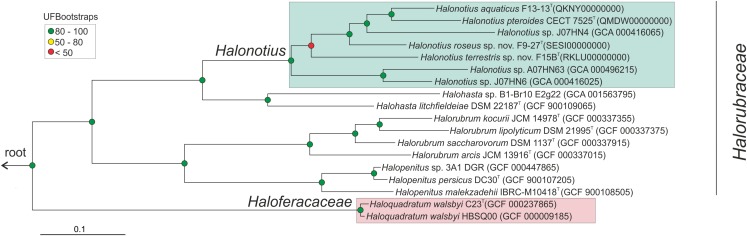
*Halonotius* phylogenomic tree. Maximum-likelihood phylogenomic tree based on the alignment of 257 shared orthologous genes. Bar, 0.1 substitution per nucleotide position. Sequence accession numbers are shown in parentheses. Red circles highlight Bootstrap/UFBootstrap values lower than 50, the yellow ones between 50 and 80 and green color is used to depict values higher than 80. The trees were rooted using the DPANN representatives *Candidatus* Nanosalinarum J07AB56 and *Candidatus* Nanosalina J07AB43.

Additional 16S rRNA gene sequences, *rpoB*′ sequences and genomes used for phylogenetic analyses were obtained from the GenBank database, and their strain designations and accession numbers are shown in Figures 1, 2 and Table 1. The trees were rooted using *Candidatus* Nanosalinarum J07AB56 and *Candidatus* Nanosalina J07AB43 as outgroups.

**TABLE 1:** Accession numbers of reference sequences used in this study.

**Name**	**16S rRNA**	***rpoB*′**	**Genome**
Strain F15B^T^	MH465135	MH454087	RKLU00000000
Strain F9-27^T^	MK478811	MK491894	SESI00000000
*Halonotius aquaticus* F13-13^T^	MH465237	QKNY00000000	QKNY00000000
*Halonotius pteroides* CECT 7525^T^	QMDW00000000	QMDW00000000	QMDW00000000
*Halonotius* sp. J07HN4	GCA 000416065	GCA 000416065	GCA 000416065
*Halonotius* sp. J07HN6	GCA 000416025	GCA 000416025	GCA 000416025
*Halonotius* sp. A07HN63	GCA 000496215	GCA 000496215	GCA 000496215
*Halohasta litchfieldiae* DSM 22187^T^	GCF 900109065	GCF 900109065	GCF 900109065
*Halopenitus persicus* DC30^T^	JF979130	GCF 900107205	GCF 900107205
*Halopenitus* sp. 3A1 DGR	GCF 000447865	GCF 000447865	GCF 000447865
*Halopenitus malekzadehii* IBRC-M 10418^T^	GCF 900108505	GCF 900108505	GCF 900108505
*Halorubrum lipolyticum* 9-3^T^/DSM 21995^T^	DQ355814	GCF 000337375	GCF 000337375
*Halorubrum saccharovorum* Ch2^T^/DSM 1137^T^	U17364	GCF 000337915	GCF 000337915
*Halorubrum arcis* JCM 13916^T^	ND	GCF 000337015	GCF 000337015
*Halorubrum kocurii* JCM 14978^T^	GCF 000337355	GCF 000337355	GCF 000337355
*Haloquadratum walsbyi* HBSQ00^T^	GCF 000009185	GCF 000009185	GCF 000009185
*Haloquadratum walsbyi* C23^T^	GCF 000237865	GCF 000237865	GCF 000237865
*Salinibacter ruber* DSM 13855^T^	ND	ND	GCF 000013045
*Spiribacter salinus* M19-40^T^	ND	ND	GCF 000319575

### Rhodopsins Tree

The genomes were scanned for the presence of rhodopsin sequences by using hmmsearch (Eddy, 2011) and a profile hidden Markov model (HMM) of the bacteriorhodopsin-like protein family (Pfam accession: PF01036). To align the highly similar identified sequences and a curated database composed of a collection of type-1 rhodopsins, MAFFT with the L-INS-i accuracy model was used (Katoh and Standley, 2013). A maximum likelihood tree with 100 bootstrap replicates was constructed based on this protein alignment, by using FastTree2 (Price et al., 2010).

### Abundance Estimation for *Euryarchaeota* and *Halonotius*

Preprocessed Illumina reads from public metagenomes were queried for putative RNA sequences by using UBLAST (Edgar, 2010) against the non-redundant SILVA (Pruesse et al., 2007) SSURef_NR99_132 database, that was clustered at 85% sequence identity by UCLUST (Edgar, 2010). Identified putative 16S rRNA sequences (*e*-value < 1e-5) were screened using SSU-ALIGN. Resulting *bona fide* 16S rRNA sequences were compared by blastn (Altschul et al., 1990) (*e*-value < 1e-5) against the curated SILVA (Pruesse et al., 2007) SSURef_NR99_132 database. Matches with identity ≥80% and alignment length ≥90 bp were considered for downstream analyses. Sequences assigned to the phylum *Euryarchaeota* and genus *Halonotius* were used to calculate the environmental abundances for these taxonomic categories.

### Fragment Recruitment

The presence of haloarchaeal strains related (at species level) to the obtained *Halonotius* strains (F15B^T^, F9-27^T^ and *Halonotius aquaticus* F13-13^T^) was assessed by performing fragment recruitments with environmental metagenomic datasets (Table 2). Briefly, in order to avoid analysis bias, we concatenated the contigs belonging to each genome and masked all the rRNA gene sequences present. Subsequently, blastn (with the cut-offs: alignment length ≥ 30 nt, identity > 95%, E value < = 1e-5) was used in order to align the metagenomic quality-filtered shotgun reads (Table 2) against the *Halonotius* genomes. Figures were constructed by using the best-hits results obtained after blastn analyses.

**TABLE 2:** Features of the different databases from hypersaline habitats analyzed in this study.

**Database name**	**Habitat**	**Salinity**	**Accession number**	**References**
SS13	Saltern	13% NaCl	SRX328504	Fernández et al., 2014a
SS19	Saltern	19% NaCl	SRX090228	Ghai et al., 2011
IC21	Saltern	21% NaCl	SRX352042	Fernández et al., 2014b
Tyrrell 0.1	Saltern	29% NaCl	SRR5637210	Podell et al., 2014
Tyrrell 0.8	Saltern	29% NaCl	SRR5637211	Podell et al., 2014
S7	Saltern	30% NaCl	SRR8921445	Unpublished
SS33	Saltern	33% NaCl	SRX347883	Fernández et al., 2014a
SS37	Saltern	37% NaCl	SRX090229	Ghai et al., 2011
Cahuill	Saltern	34% NaCl	SRX680116	Plominsky et al., 2014
Gujarat	Saline soil	ND	ERP005612	Patel et al., 2015
SMO1	Saline soil	24.0 dS/m	SRR5753725	Vera-Gargallo et al., 2018
SMO2	Saline soil	54.4 dS/m	SRR5753724	Vera-Gargallo et al., 2018

### Identification of B_12_-Dependent Enzymes in Metagenomic Datasets

**FIGURE 3:**
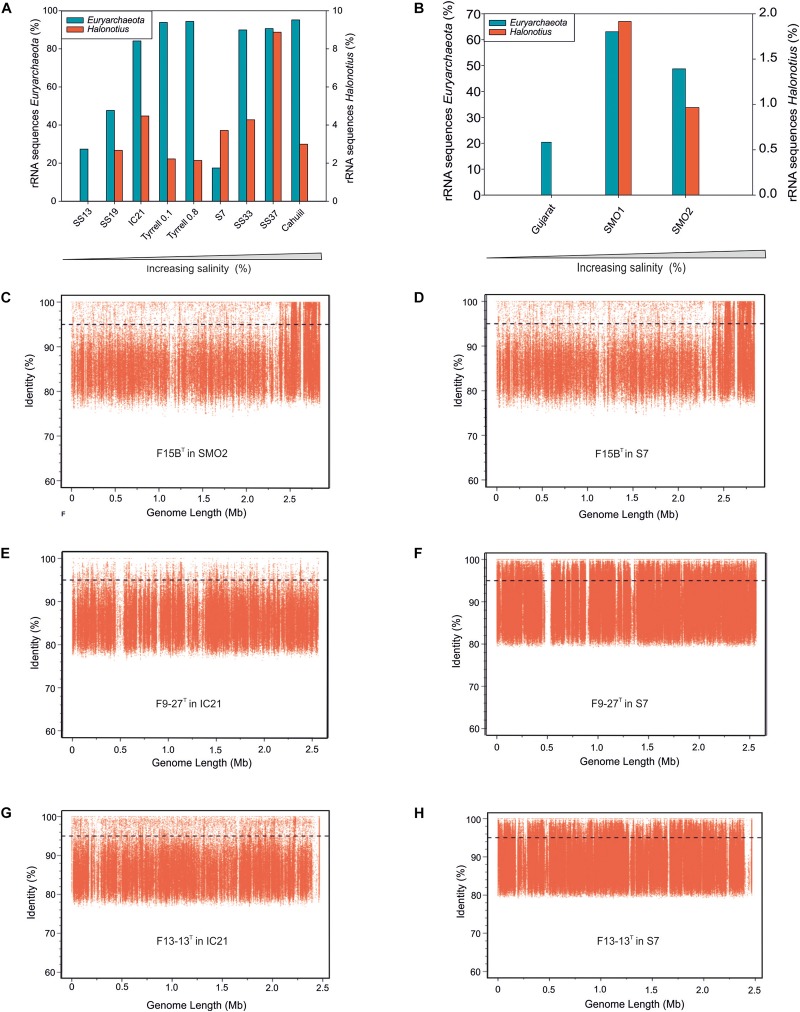
*Halonotius* sequences abundance. **(A,B)** Percentage of rRNA sequences related to *Euryarchaeota* and *Halonotius* recovered from 12 shotgun metagenomes. The metagenomic datasets are ordered by salinity gradient. **(C–H)** Recruitment plots of *Halonotius* strains F15B^T^, F9-27^T^ and *Halonotius aquaticus* F13-13^T^ against different hypersaline habitats. In each panel the *Y* axis represents the identity percentage and *X* axis represents the genome length. A restrictive cut-off 95% of nucleotide identity in at least 30 bp of the metagenomic read was used. The black dashed line shows the threshold for presence of same species (95% identity). SS13, Metagenome from Santa Pola solar saltern (Alicante, Spain), 13% salinity (SRX328504); SS19, Metagenome from Santa Pola solar saltern (Alicante, Spain), 19% salinity (SRX090228); IC21, Metagenome from Isla Cristina solar saltern (Huelva, Spain), 21% salinity (SRX352042); Tyrrell 0.1, Metagenome from Lake Tyrrell (Victoria, Australia), 29% salinity (SRR5637210); Tyrrell 0.8, Metagenome from Lake Tyrrell (Victoria, Australia), 29% salinity (SRR5637211); S7, Metagenome from Fara Fund hypersaline meromictic lake, 30% salinity (SRR8921445); SS33, Metagenome from Santa Pola solar saltern (Alicante, Spain), 33% salinity (SRX347883); SS37, Metagenome from Santa Pola solar saltern (Alicante, Spain), 37% salinity (SRX090229); Cahuill, Metagenome from Cahuil lagoon (Chile), 34% salinity (SRX680116); Gujarat, Metagenome from Little Rann of Kutch hypersaline soil (Gujarat, India), (ERP005612); SMO1, Metagenome from Marismas del Odiel Salt Marshes hypersaline soil (Huelva, Spain), 24 mS/cm salinity (SRR5753725); SMO2, Metagenome from Marismas del Odiel Salt Marshes hypersaline soil (Huelva, Spain), 54 mS/cm salinity (SRR5753724).

Three metagenomic datasets (i.e., IC21, SMO2 and S7; Table 2) were selected for B_12_-dependent enzyme screening, based on the results of the fragment recruitment analyses (Figure 3). Preprocessing of raw sequencing reads was performed as described above. MEGAHIT software (v1.1.5) (Li et al., 2015) with the k-mer sizes: 396 49,69,89,109,129,149, and default settings was used for metagenomic assembly. Protein-coding genes were predicted by MetaProdigal (Hyatt et al., 2012). hmmsearch (-E -evalue 1e-5 -prcov 50 -hmcov 50) (Eddy, 2011) was used for identification of B_12_-dependent enzymes in the assembled metagenomic datasets. The identified protein sequences and the used HMMs are deposited in figshare^1^.

### ANI, AAI, DDH and Protein Isoelectric Points

Average nucleotide identity (ANI) and average amino acid identity (AAI) were calculated as previously described by Konstantinidis and Tiedje (2005a, b), respectively. Digital DDH values were determined using the Genome-to-Genome Distance Calculator (GGDC) website using formula 2 as described by Auch et al. (2010) and Meier-Kolthoff et al. (2013). The isoelectric points of proteins were calculated using the iep program implemented in the EMBOSS package (Rice et al., 2000).

### Chemotaxonomic Analysis

Cultures of the strains F15B^T^, F9-27^T^, *Halonotius aquaticus* F13-13^T^ and *Halonotius pteroides* CECT 7525^T^ [obtained after 14 days of aerobic incubation in R2A with 25% (w/v) salts liquid medium under optimal conditions] were used for chemotaxonomic analyses. For lipid identification *Halobacterium salinarum* DSM 3754^T^ and *Halorubrum saccharovorum* DSM 1137^T^ were used as reference. The lipids of these two species have been previously experimentally identified (Kushwaha et al., 1982; Torreblanca et al., 1986). The strains used for comparisons were cultured according to the original descriptions for each species and standardized to the same incubation conditions. Lipid extraction was carried out following the method described by Corcelli and Lobasso (2006). Total lipid extracts were analyzed by high-performance TLC using HPTLC silica gel 60 plates crystal back (10 × 20 cm; Merck art. 5626); the plates were eluted in the solvent system chloroform/methanol/90% acetic acid (65: 4: 35, by vol.). For detection of all lipids, phospholipids and polar lipids, the plate was sprayed with sulfuric acid 5% in water, molybdenum blue spray reagent and phosphomolybdic acid, respectively and compared with the ones from *Halobacterium salinarum* DSM 3754^T^ and *Halorubrum saccharovorum* DSM 1137^T^.

### Phenotypic Characterization

Phenotypic tests for strains F15B^T^ and F9-27^T^ and *Halonotius aquaticus* F13-13^T^ and *Halonotius pteroides* CECT 7525^T^, were performed according to the minimal standards delineated for the taxonomic description of novel taxa of the *Halobacteria* (Oren et al., 1997), following the same methodology previously described (Durán-Viseras et al., 2019).

## Results and Discussion

### Phylogenetic Analyses

After extensive haloarchaeal strains isolations from two hypersaline environments located in South west Spain (Odiel Saltmarshes and Isla Cristina), the strains F15B^T^ and F9-27^T^ were selected for further analyses. Both strains proved to be closely related to members of the genus *Halonotius*, as shown by 16S rRNA gene sequence comparison with available sequences in the public databases and by phylogenetic reconstruction (Figure 1A). While the 16S rRNA gene sequence of strain F15B^T^ (1400 bp) was most closely related to *Halonotius aquaticus* F13-13^T^ (96.4% sequence similarity), strain F9-27^T^ (1394 bp) was more similar to *Halonotius pteroides* CECT 7525^T^ (96.9% sequence similarity). The 16S rRNA gene sequence similarity between strains F15B^T^ and F9-27^T^ was 95.8%. The 16S rRNA sequence obtained by PCR and the one extracted from the genome for strains F15B^T^ and F9-27^T^ were compared, and proved to be identical. The 16S rRNA gene sequence similarity to other genera such as *Halohasta* or *Halorubrum*, was lower than 94 and 87%, respectively. The maximum-likelihood 16S rRNA phylogenetic tree showed that strains F15B^T^ and F9-27^T^ grouped within the *Halonotius* cluster, but in independent branches from *Halonotius pteroides* CECT 7525^T^ and *Halonotius aquaticus* F13-13^T^, respectively, suggesting that they could constitute different species within the genus *Halonotius* (Figure 1A).

Limitations of the 16S rRNA gene sequence analysis to discriminate species of haloarchaea have been reported (Boucher et al., 2004; Sun et al., 2013), and thus a phylogenetic tree based on the comparison of the *rpoB’* gene sequences was reconstructed (Figure 1B). In concordance to the 16S rRNA tree, both strains clustered with species of the genus *Halonotius*, but were located in different branches (Figure 1B). Strain F15B^T^ (1827 bp) showed a nucleotide similarity of 89.1% with *Halonotius aquaticus* F13-13^T^ and strain F9-27^T^ (1827 bp) shows a nucleotide similarity of 92.0% with *Halonotius* sp. J07HN4 and 91.8% with *Halonotius aquaticus* F13-13^T^. The *rpoB′* gene sequence similarity between strains F15B^T^ and F9-27^T^ was 89.3%.

### Genome Sequence Characteristics

The genomes of the isolated strains, F15B^T^ and F9-27^T^ were successfully sequenced, and in conjunction with those of the two currently described species of the genus, *Halonotius pteroides* CECT 7525^T^ and *Halonotius aquaticus* F13-13^T^, were assembled *de novo*. The characteristics of all these genomes are shown in Table 3. As most members of the class *Halobacteria* (phylum *Euryarchaeota*), all genomes had a high G + C content, between 59.5–62.7 mol%, with F9-27^T^ having the highest. All four *Halonotius* strains studied have similar genome size, ranging from 2.5–3.0 Mb, making them the smallest ones reported for members of the family *Halorubraceae*. Additional genomic features are shown in Supplementary Figure 1.

**TABLE 3:** General features of sequenced genomes.

	**Strain F15B^T^**	**Strain F9-27^T^**	***Halonotius aquaticus* F13-13^T^**	***Halonotius pteroides* CECT 7525^T^**
Size (Mb)	2.9	2.6	2.5	3.0
Contigs	26	4	10	41
Completeness (%)	98.4	99.4	99.2	99.6
G + C (mol%)	61.5	62.7	61.2	59.5
Protein coding genes	3067	2686	2568	3092
rRNA	5	2	3	4
tRNA	47	44	45	45
Accession number	RKLU 00000000	SESI 00000000	QKNY0 0000000	QMDW0 0000000

### Phylogenomic Analysis

To confirm the phylogenomic relationships previously obtained by 16S rRNA and *rpoB*′ genes sequence comparison between *Halonotius* strains, *Halonotius* metagenome-assembled genomes (MAGs) and other related haloarchaeal species, a phylogenomic tree based on core orthologous genes was constructed. *Candidatus* Nanosalina J07AB43 (GCA_000220375) and *Candidatus* Nanosalinarum J07AB56 (GCA_000220355) MAGs were used as outgroups. A total of 257 core orthologous genes were shared between all genomes. The phylogenomic tree based on the concatenated alignment, showed that all *Halonotius* genomes clustered together in a single branch, well separated from other haloarchaeal groups and supported by high bootstrap values (Figure 2). Additionally, also supported by high bootstrap values, within the *Halonotius* cluster, all *Halonotius* strains appeared monophyletically separated, suggesting they represent different species within this genus. The results are in concordance with the initial phylogeny described by 16S rRNA and *rpoB*′ genes sequence comparison, which suggest that for this genus both genes could be used as reliable phylogenetical markers.

### Ecological Distribution and Abundance of the Genus *Halonotius*

Metagenomic studies carried out in hypersaline environments have enabled the determination of the microbial diversity of these habitats, suggesting that a large proportion of haloarchaeal members, abundant in these systems, had not been isolated or characterized before (Ventosa et al., 2015). Based on 16S rRNA gene sequence reads abundances from different aquatic and terrestrial hypersaline systems with different salinities (see Materials and Methods), representatives of the genus *Halonotius* were found to be present in almost all metagenomes from habitats with intermediate to high salinity concentrations. The genus *Halonotius* comprised up to 9% of the prokaryotic community in aquatic saline environments (Figure 3A) and up to 1.8% of the prokaryotic community in saline soils (Figure 3B). The data confirm that members of the genus *Halonotius* are abundant microorganisms in hypersaline environments, especially at high salinities (from 19 to 37% total salts) but are completely absent in habitats without or with lower salt concentrations. In this sense, we can consider them as obligately extreme halophiles that prefer habitats with high salinities.

To assess the distribution of the cultured *Halonotius* strains, fragment recruitment was carried out against all those metagenomic datasets mentioned above. The metagenomes which provided significant recruitment values are shown in Figures 3C–H. All *Halonotius* strains used in this study showed high recruitment values in their environment of origin: the metagenomic dataset SMO2 reported from the Odiel Saltmarshes saline soil in Huelva, Spain for strain F15B^T^, and dataset IC21 obtained from Isla Cristina salterns in Huelva, Spain, for strains F13-13^T^ and F9-27^T^, where strain F13-13^T^ seemed to be more abundant. Remarkably, highest recruitment values were obtained against the metagenomic dataset S7, which corresponds to a hypersaline meromictic pit lake located in Transylvanian Basin, Romania; suggesting a worldwide distribution for this genus. Even the highest abundance of 16S rRNA *Halonotius* reads was obtained in SS37 metagenome, none of the *Halonotius* isolates recruited significantly in that metagenome, only *Halonotius pteroides* CECT 7525^T^ seemed to be present in that metagenome dataset (Supplementary Figure 2). The abundance of reads above 95% similarity for *Halonotius pteroides* CECT 7525^T^ against metagenomic database SS37 and the above mentioned 16S rRNA reads abundance in this metagenome, indicates that there might be other *Halonotius* species present in significant amounts in this specific habitat.

Furthermore, to evaluate the presence of *Halonotius* worldwide, we have searched the SILVA database for 16S rRNA sequences belonging to this genus. Results indicate that members of the genus *Halonotius* are found in a variety of hypersaline environments from different countries, such as: Canada, United States, Bolivia, Tunisia, Spain, Romania, Turkey, Russia, China, and Australia (Supplementary Figure 3). All these data, in addition to the 16S rRNA reads abundance along the different metagenomes, support a worldwide distribution of this genus.

### Metabolism

**FIGURE 4:**
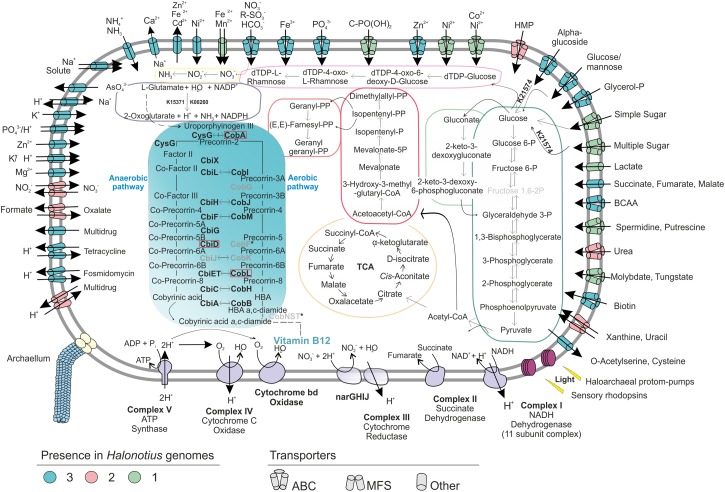
Metabolic reconstruction of the isolated strains *Halonotius terrestris* sp. nov. F15B^T^, *Halonotius roseus* sp. nov. F9-27^T^ and *Halonotius aquaticus* F13-13^T^. The turquoise panel depicts the simplified metabolic pathway of vitamin B_12_ biosynthesis; the gene names highlighted in boldface were present in the genomes, while the ones in gray are inferred to be absent in archaea. Horizontal arrows indicate homology between aerobic and anaerobic pathway enzymes. The discontinuous lines correspond to complete pathways not represented in the figure. The dark green box highlights glycolysis (Embden–Meyerhof–Parnas), while the light green one shows the Entner–Doudoroff pathway modified for haloarchaea. The orange depicts the tricarboxylic acid cycle (TCA); pink color corresponds to isoprenoid biosynthesis: C5 isoprenoid (dark) and C10–C20 isoprenoid (light). The light violet box corresponds to dTDP-L-rhamnose biosynthesis; the yellow color shows the assimilatory nitrate reduction pathway; dark purple is used for the L-glutamate degradation by glutamate dehydrogenase (GdhA). Alpha-amylase enzyme for glucose degradation is also represented by its KO identifier. Transparency was used to show the inferred components of the complex III of electron transport chain. Red boxes are used for genes not present in the studied genomes. BCAA, branched-chain amino acid; HMP, hydroxymethylpyrimidine; TCA, tricarboxylic acid cycle.

*Halonotius* strains were isolated on complex media for heterotrophic aerobic microbes (see Materials and Methods). Genome annotation of *Halonotius* members confirm their heterotrophic metabolism. No evidences for photosynthetic or chemolithotrophic capabilities were found in any of these genomes. In line with their heterotrophic capabilities, several ABC and other transporters for uptake of carbohydrates were identified, forming a large fraction of the genes in all *Halonotius* genomes (Figure 4).

Representative genes for central carbohydrate metabolism like the tricarboxylic acid cycle, pentose phosphate, gluconeogenesis and Entner–Doudoroff pathway were present (Figure 4). For the oxidation of pyruvate to acetyl CoA (Figure 4), only the anaerobic route via pyruvate ferredoxin oxidoreductase (*porA* and *porB*) seemed to be present, while the aerobic route via pyruvate dehydrogenase (PDH) was absent. Additionally, genes encoding an α-amylase (K21574), which can degrade complex carbohydrates to glucose were detected (Figure 4). Remarkably, in agreement with previous studies on haloarchaea (Falb et al., 2008; Anderson et al., 2011), the standard Embden-Meyerhof pathway of glycolysis appears to be incomplete, with 6-phosphofructokinase (the key enzyme of the classical pathway), found absent in all studied genomes (Figure 4). This suggest that haloarchaea could utilize the Entner-Doudoroff pathway for glucose degradation or that alternative enzymes could be used for this step.

Regarding nitrogen metabolism, *Halonotius* genomes encode the essential genes involved in the assimilation of ammonia and amino acid metabolism, glutamine synthetase and glutamate synthase. Accordingly, all genomes also presented the gene encoding the high-affinity ammonium transporter Amt, indicating that nitrogen uptake occurs in its most reduced form, ammonia (Figure 4). Along similar lines, strains F15B^T^ and F9-27^T^ showed a major facilitator superfamily (MFS) antiporter for nitrate/nitrite uptake (Figure 4). Genes encoding the enzymes for nitrate reduction assimilation to ammonia, nitrate reductase and nitrite reductase, were also present. Besides, several ABC transporters for nitrogen sources, such as branched-chain amino acids, urea, putrescine/spermidine and nitrate-nitrite/taurine were also found (Figure 4). In addition, *Halonotius* genomes showed other genes which could provide nitrogen rich compounds, by degradation of amino acids, such as L-glutamate, to ammonia generating NADPH (glutamate dehydrogenase) (Figure 4) or by urea degradation, which was only absent in strain F15B^T^. Most pathways for amino acid biosynthesis were also found in the studied genomes (Supplementary Table 1).

The presence of the high-affinity phosphate transport system (PstSCAB), suggests that this is the main route of uptake of inorganic phosphate (Figure 4). All studied genomes of *Halonotius* also contained the ABC transporter, PhnCDE, a high affinity uptake system for phosphonates (Figure 4), which are organophosphorous compounds with a carbon–phosphorous bond that under phosphate starvation conditions could be used also as a nutritional source of phosphorous (Villarreal-Chiu et al., 2012).

In addition, several antimicrobial compounds and multidrugs transport systems were found and genes encoding archaella were also discovered in all *Halonotius* genomes.

### Cobalamin (Vitamin B_12_) Biosynthesis

**FIGURE 5:**
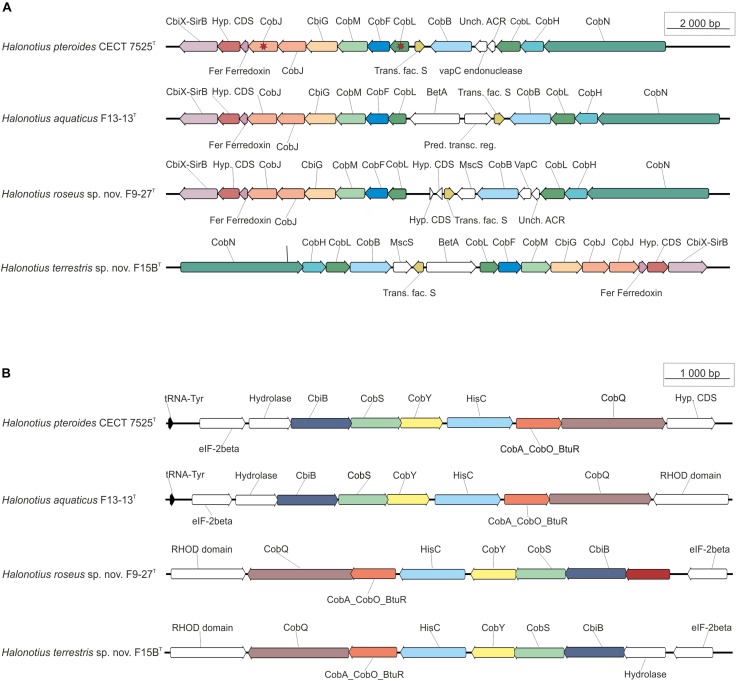
*Halonotius* cobalamin synthesis gene clusters involved in cobalamin synthesis. The figure highlights the co-occurrence of cobalamin synthesis genes and their conserved order in the analyzed *Halonotius* genomes (*n* = 4). **(A)** Represents the genes from the first stage of the pathway, from uroporphyrinogen III to *a,c*-diamine. **(B)** Represents the genes from the second stage of the pathway, from *a,c*-diamine to vitamin B_12_. The red star marks genes that are present in two copies. The genes depicted in white color were found not to be conserved in the gene cluster. The scale bar indicates gene lengths (in bp).

Genome-scale metabolic reconstructions performed for the *Halonotius* members brought to light the complete cobalamin biosynthesis pathway (Figures 4, 5), suggesting the genetic capability of the strains of this genus for its *de novo* synthesis. Cobalamin (vitamin B_12_) is a complex metabolite, produced only by a subset of bacteria and archaea, and essential cofactor required by many organisms from all the domains of life. Cobalamin can be synthesized by either an aerobic or anaerobic pathway from uroporphyrinogen III as precursor, involving 20 enzymatic steps. Several enzymes are homologous and shared by both pathways, while only few are specifically oxygen-requiring or oxygen-sensitive (Moore and Warren, 2012). The complex structure of cobalamin and the high metabolic toll of its biosynthesis pathway (Morris, 2012; Doxey et al., 2015) fueled a ‘race’ toward a genomic removal, thus designating the few taxa capable of its biosynthesis as ‘losers’ that perform a Black Queen function (Morris, 2015). This term alludes to the game of Hearts, where players try to avoid being stuck with the queen of spades, and has been used to describe costly genes or functions that members of the community benefit from losing. Thus, generating individual “beneficiaries” of reduced genomic content depending on luckless “helpers” that provide goods to the whole community (Morris et al., 2012). The role of representatives of the Archaea in cobalamin production has been poorly studied and limited to *Thaumarchaeota*, halophilic *Crenarchaeaota* and marine methanogenic *Euryarchaeota* (Rodionov et al., 2003; Doxey et al., 2015).

*Halonotius* genomes contained almost all genes involved in aerobic and anaerobic pathways, with only a few gaps (i.e., *CobG* and *CobK* were missing from the aerobic pathway; *CbiD* and *CbiJ* were missing from the anaerobic pathway) (Figure 4). Additionally, all genes encoding the conversion of cobyrinic acid *a,c*-diamide to vitamin B_12_ were also found and are represented by a discontinuous line on the metabolic map (Figures 4, 5B). None of the absent genes were either detected in any known archaeal cobalamin producers and therefore should not be considered as essential genes for this pathway (Doxey et al., 2015). Furthermore, previous studies considered the presence of *cbiA*/*cobB*, *cbiC*/*cobH* or *cobT* genes in a microorganism, as reliable indicators for the complete pathway (Bertrand et al., 2011). As far as the authors are aware, despite a previous report on *Halobacterium salinarum* NRC-1 cobamide production (Woodson et al., 2003), little is known about the cobalamin biosynthesis pathway in hypersaline environments, or halophilic archaea at large. Due to the detection of the cobalamin biosynthesis genes clusters in all the analyzed *Halonotius* genomes (Figure 5), and the widespread distribution and abundance (Figure 3 and Supplementary Figure 3) of this genus in hypersaline environments, we hypothesize that these organisms perform a critical role in the microbial community, where they contribute to the maintenance of the environmental cobalamin supply and modulate trophic interactions. Moreover, the detection of B_12_-dependent enzymes (e.g., methylmalonyl-CoA mutases; metH, MTR 5-methyltetrahydrofolate-homocysteine methyltransferases; nrdA/nrdE ribonucleoside-diphosphate reductases; pduC propanediol dehydratases) (Supplementary Table 2) in metagenomic datasets where *Halonotius* genomes were inferred to be present, pinpoints toward metabolic dependencies between the cobalamin suppliers and consumers.

### Osmoregulation

The mechanisms used by microorganisms to deal with their cellular adjustment to high salinity environments are diverse and can be classified in *salt-in* and *salt-out* strategies (Galinski and Trüper, 1994; Wood et al., 2001), physiological osmostress adaptations which are not necessarily mutually exclusive (Deole et al., 2013; Oren, 2013; Youssef et al., 2014). The osmotic balance in microorganisms that use the *salt-in* strategy is achieved by accumulation of a large fraction of inorganic ions in the medium, as K^+^ and Cl^–^ via transport (Oren, 2011), while Na^+^ ions are excluded as much as possible from cells (Gunde-Cimerman et al., 2018). However, in order to keep the proteins solubility and to ensure the functionality of key cellular activities, the entire proteome of the microorganism required compensatory changes for the cytoplasm′s adaptation to the high ionic strength (Oren, 2013; Saum et al., 2013; Talon et al., 2014; Warden et al., 2015). This has left an acidic signature on the proteome of those *salt-in* microorganisms, reducing its surface hydrophobicity and limiting these microorganisms to hypersaline habitats where the external salinity does not frequently fluctuate.

**FIGURE 6:**
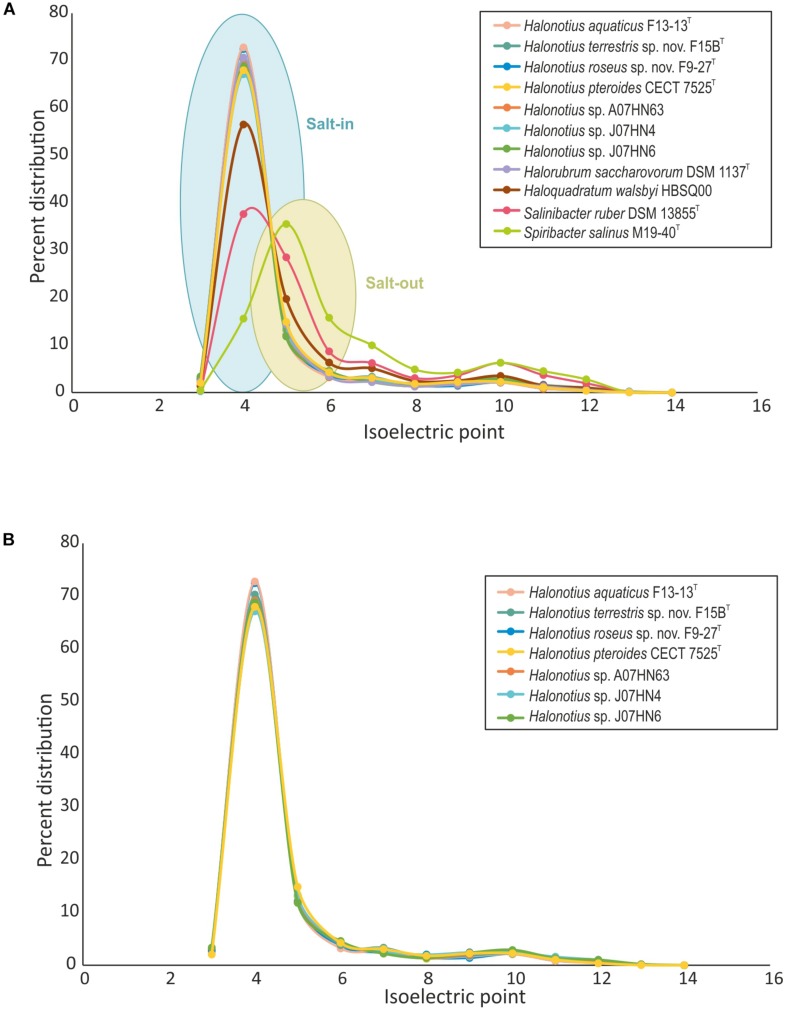
Comparison of isoelectric profiles of *Halonotius* proteomes with those of other prokaryotic species. **(A)** Comparison of isoelectric point of predicted proteins for *Halonotius* and other prokaryotic species, computed for each translated genome and shown as a percentage of distribution. **(B)** Comparison of isoelectric point of predicted proteins between proteomes of cultured *Halonotius* genomes and environmental *Halonotius* MAGs, shown as a percentage of distribution.

We have characterized the proteome of all available *Halonotius* genomes (Figure 6), and to analyze the differences in protein acidity we have compared them to proteomes from *salt-in* halophilic archaea [*Haloquadratum walsbyi* HBSQ00 (Bolhuis et al., 2006) and *Halorubrum saccharovorum* DSM 1137^T^ (Becker et al., 2014)], a *salt-in* halophilic bacterium [*Salinibacter ruber* DSM 13855^T^ (Mongodin et al., 2005)] and to that of a *salt-out* bacterium [*Spiribacter salinus* M19-40^T^ (López-Pérez et al., 2013)] (Figure 6). The proteome pI plot showed a single peak around 4.0 for all haloarchaeal genomes and for *Salinibacter ruber* DSM 13855^T^, which follow the trend of amino acid use in the hypersaline system (Fernández et al., 2014b) being acidic residues more frequently employed and thus suggesting a *salt-in* strategy for *Halonotius* strains. Only *Spiribacter salinus* M19-40^T^, microorganism employing a *salt-out* strategy, resulted in a different single peak around 4.5.

Another method to determine the salt adaptation of a microorganism is the reliance on the Na^+^ gradient to transport actively nutrients into the cell (Oren, 2011; Gunde-Cimerman et al., 2018). All *Halonotius* strains have several secondary transporters that catalyze the translocation of solutes across the cytoplasmic membrane using electrochemical ion gradients. Na^+^ is extruded from the cells by Na^+^/H^+^ and Na^+^/Ca^2+^ antiporters systems while K^+^ can enter the cells passively through K^+^ channels in the membrane and by K^+^/H^+^ symporters systems (Figure 4), consistent with *salt-in* adaptations to a hypersaline environment. The necessary energy is derived from the proton gradient over the membrane, generated by respiratory electron transport and from the light-dependent proton pumping rhodopsin (Figure 4).

In contrast, the *salt-out* strategy is based in keeping most of inorganic ions out and using organic compatible solutes to balance the high salinity of the external environment (Empadinhas and Da Costa, 2008; Gunde-Cimerman et al., 2018). In the case of *Halonotius*, ABC-type transport systems or other transporters for compatible solutes uptaking were not observed in their genomes. In the same way, none of the genes encoding key enzymes for the synthesis of organic compatible solutes, such as glycine betaine, ectoine, hydroxyectoine, proline, biphosphoglycerate or trehalose were found in any of the studied *Halonotius* genomes. This suggests that members of the genus *Halonotius* do not use this mechanism to balance the high salinity of the environment.

### Rhodopsin Analysis

Microbial rhodopsins are a family of photoactive retinylidene proteins widely distributed within the microbial world. They were first discovered in haloarchaea in the early’s 1970 (Oesterhelt and Stoecknius, 1971; Oesterhelt and Stoeckenius, 1973), however, later genomic and metagenomic sequencing revealed homologs in many disparate eukaryotes and bacteria, and later in the marine group II euryarchaeota (Frigaard et al., 2006). These kind of proteins are characterized by their diversity of function, using variations of a shared seven-transmembrane helix structure and similar photochemical reactions to carry out distinctly different light-driven energy and sensory traduction processes (Govorunova et al., 2017). Their biological functions fall into two categories: (a) photoenergy transducers that convert light into electrochemical potential to energize cells (light-driven ion pumps), which catalyze outward active transport of protons, and (b) photosensory receptors, that use light to gain information about the environment to regulate cell processes (Govorunova et al., 2017).

**FIGURE 7:**
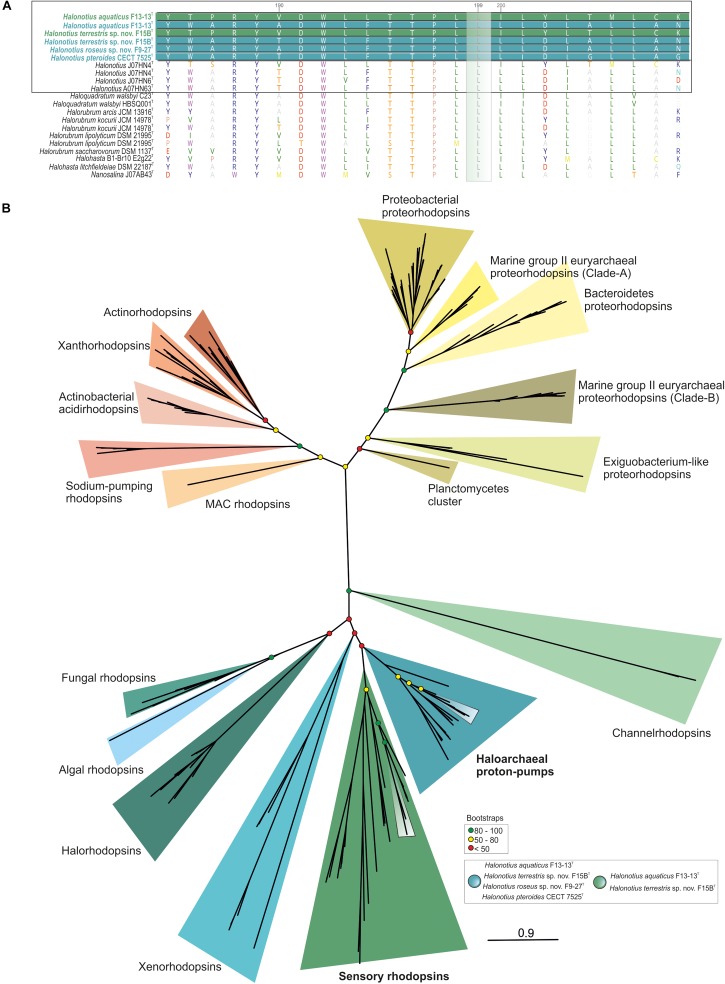
*Halonotius* rhodopsin tree. **(A)** Alignment comparison of rhodopsin protein sequence from *Halonotius* and other haloarchaea. Sequence accession numbers are shown in parentheses. Cultured *Halonotius* sequences are highlighted in boldface; blue corresponds to haloarchaeal proton-pump rhodopsins, while green highlights the sensory ones. The green box shows the position 199 of the rhodopsin alignment. The leucine (L) variant absorbs maximally in the green spectrum. **(B)** Maximum-likelihood phylogenetic tree constructed using 220 rhodopsins sequences. Boxes were used to highlight rhodopsin sequences belonging to *Halonotius* (see box at bottom right side of the figure). Bootstrap values on nodes are indicated by colored circles. Red circles show values lower than 50, the yellow ones between 50 and 80; green color is used to depict values higher than 80.

Rhodopsin-like sequences were found in all members of the genus *Halonotius* (Figure 7B). Specifically, haloarchaeal proton-pumps were found in all sequenced *Halonotius* genomes, which indicates that ATP synthesis light-mediated is a typical feature of species of this genus. Additionally, strains F13-13^T^ and F15B^T^ also encoded sensory rhodopsins, suggesting additional metabolic flexibility in illuminated conditions for these two strains.

According to major absorption light wavelength, rhodopsins could be classified as “green-absorbing” and “blue-absorbing.” A single amino acid residue determines this preference. The leucine (L) and methionine (M) variants absorb maximally in the green spectrum while the glutamine (Q) variant absorbs maximally in the blue spectrum (Man et al., 2003). Alignment of rhodopsins sequences found in *Halonotius* genomes showed a leucine amino acid in this position, indicating that they show a green light absorption (Figure 7A).

### Amino Acid Identity (AAI), Average Nucleotide Identity (ANI) and Digital DNA-DNA Hybridization (DDH)

**FIGURE 8:**
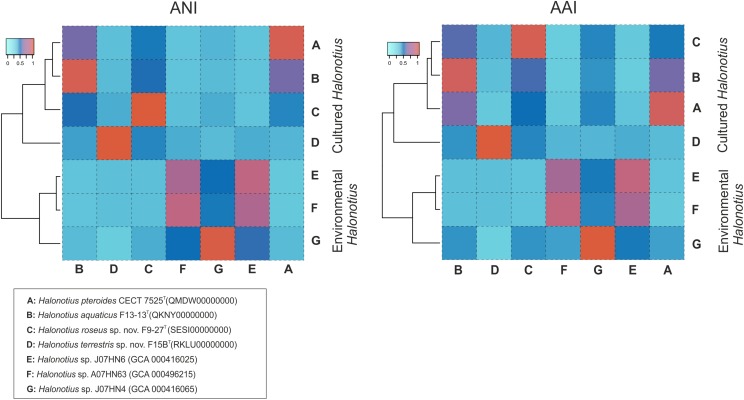
Amino acid identities and average nucleotide identities between *Halonotius* genomes. Hierarchical clustering relationships between cultured *Halonotius* genomes and environmental *Halonotius* MAGs. Amino acid identities (AAI) and average nucleotide identities (ANI) are represented by heat maps, where similarity values are represented by the color key histograms on the upper panels. Strains and sequence accession numbers are shown in the box at the bottom of the figure.

The AAI, the ANI, and the digital DNA–DNA hybridization (DDH) parameters between strains F15B^T^, F9-27^T^, *Halonotius pteroides* CECT 7525^T^, *Halonotius aquaticus* F13-13^T^ and other environmental *Halonotius* MAGs, were calculated as described in the “Materials and Methods” and the results are shown in Figure 8 and in Table 4. A threshold AAI value of 65% has been established for genus delineation (Klappenbach et al., 2007; Konstantinidis et al., 2017). AAI values for all *Halonotius* genomes were in all cases higher than 75% (Figure 8), indicating that all of them belongs to the genus *Halonotius*.

**TABLE 4:** ANI and DDH values between genomes of *Halonotius*.

**ANI digital DDH**	**1**	**2**	**3**	**4**	**5**	**6**	**7**
(1) Strain F15B^T^	–	81.8	81.0	79.7	78.9	80.3	80.3
(2) Strain F9-27^T^	33.3	–	85.2	83.8	82.2	80.0	80.0
(3) *Halonotius aquaticus* F13-13^T^	24.7	29.6	–	88.2	81.0	79.4	79.3
(4) *Halonotius pteroides* CECT 7525^T^	23.4	27.8	35.7	–	80.9	79.1	79.1
(5) *Halonotius* sp. J07HN4	22.7	25.6	24.4	24.7	–	86.1	84.9
(6) *Halonotius* sp. J07HN6	23.9	24.6	24.0	23.9	39.2	–	96.6
(7) *Halonotius* sp. A07HN63	24.0	24.6	23.8	23.9	36.5	71.1	–

For species delineation, a threshold ANI value of 95–96% (Konstantinidis and Tiedje, 2004; Richter and Rosselló-Móra, 2009) and a DDH value of 70% (Stackebrandt and Goebel, 1994; Auch et al., 2010) has been established. ANI values for all four strains of *Halonotius* studied were in all cases lower than 88% (Figure 8 and Table 4) and DDH values lower than 35.7% (Table 4), suggesting that they constitute four separate taxa at the species level. We can conclude that strains F9-27^T^ and F15B^T^ represent two different species of the genus *Halonotius*, for which we propose the new designations *Halonotius roseus* sp. nov. and *Halonotius terrestris* sp. nov., respectively.

### Phenotypic Characterization

The different phenotypic features between strains F9-27^T^ and F15B^T^ and the other members of the genus *Halonotius* are shown in Table 5. More phenotypic features of strains F9-27^T^ and F15B^T^ are detailed in their species descriptions given below.

**TABLE 5:** Characteristics that differentiates *Halonotius terrestris* F15B^T^ and *Halonotius roseus* F9-27^T^ from related species of the genus *Halonotius*.

**Characteristics**	**1**	**2**	**3**	**4**
Cell morphology	Short rods and pleomorphic shape	Pleomorphic shape	Long and curved rods, sometimes associated in pairs	Flattened rods^∗^
Cell size (μm)	1.1 × 1.7	2.8 × 1.1	1.1–4.4 × 0.5	0.7–1.5 × 2–6^∗^
Cell motility	+	–	+	+^∗^
Colony size (mm)	0.2	0.1	0.3–0.4	0.5–1.0^∗^
NaCl (%, w/v) range	15–30	15–30	15–30	16–36^∗^
NaCl (%, w/v) optimum	25	25	25	18^∗^
Temperature range for growth (°C)	25–50	25–50	28–50	25–45^∗^
Optimum temperature for growth (°C)	37	37	37	45
pH range	7.0–7.5	7.0–9.0	6.0–8.5	5.0–8.5^∗^
pH optimum	7.0	7.5	7.5	7.5
Anaerobic growth:				
L-arginine	–	–	+	–
Potassium nitrate	–	–	–	w
Dimethyl sulfoxide	–	–	+	w
Nitrite reduction	+	–	–	–
Methyl red test	+	–	w	w
H_2_S production	–	+	–	–
Production of acids from:				
D-amygdalin	–	–	+	–
D, L-arabinose	w	–	+	w
Arbutin	–	–	–	w
D-fructose	w	–	+	–
D-glucose	–	–	w	–
Glycerol	–	–	–	+
Sorbitol	–	–	–	w
D-xylose	+	–	+	+
Utilization as sole carbon and energy source:				
L-alanine	+	–	–	–
L-arginine	+	–	–	–
Butanol	–	w	–	+
Citrate	–	–	+	–
D-galactose	+	–	–	–
D-glucose	+	–	+	+
Glycerol	+	–	+	+
Isoleucine	–	–	–	+
D-mannitol	+	–	–	–
Maltose	+	–	–	–
Methanol	–	–	–	+
Pyruvate	–	–	–	+
L-raffinose	+	–	+	–
D-sorbitol	–	–	–	+
Sucrose	–	+	–	–
D-xylose	–	w	–	–
DNA G + C content (mol%, genome)	61.5	62.7	61.2	59.7

*Strains: 1, *Halonotius terrestris* F15B^*T*^; 2, *Halonotius roseus* F9-27^*T*^; 3, *Halonotius aquaticus* F13-13^*T*^; 4, *Halonotius pteroides* CECT 7525^*T*^. All data from this study unless otherwise indicated. +, positive; –, negative; w, weakly positive. ^∗^Data from Burns et al. (2010).*

### Chemotaxonomic Characterization

An exhaustive comparative analysis of the total lipids, phospholipids and polar lipids, between strains F9-27^T^ and F15B^T^ and the previously described *Halonotius* species, was carried out (Supplementary Figure 4). Results revealed that all of them possess phosphatidylglycerol (PG), phosphatidylglycerol phosphate methyl ester (PGP-Me), phosphatidylglycerol sulfate (PGS) and one glycolipid chromatographically identical to sulfated diglycosil diether (S-DGD-1) as major polar lipids. As the presence of PGS was not reported at the description of the genus *Halonotius* and *Halonotius pteroides* (Burns et al., 2010), here we also propose the emended description of this genus in order to include this and other features of species of the genus.

## Conclusion

This study provides a complete comparative genomic analysis of the genus *Halonotius*, showing evidence of the ubiquity and abundance of species of this genus in different hypersaline environments and significantly expanding our knowledge of this almost unknown haloarchaeal genus. Results from *Halonotius* 16S rRNA read abundances from different aquatic and terrestrial hypersaline systems and *Halonotius* genomes recruitments against these systems, confirm *Halonotius* abundance in these environments. Remarkably, results from the complete metabolic analysis of this genus brought to light that *Halonotius* members present the complete cobalamin biosynthesis cluster, suggesting their capability to produce cobalamin. Thus, we suggest that species of this genus could play a relevant environmental role for the community in *de novo* cobalamin synthesis on these saline environments, and thus, the members of *Halonotius* could perform a Black Queen function among members of the haloarchaea in hypersaline habitats (Morris et al., 2012; Mas et al., 2016).

Additionally, this study indicates that members of the genus *Halonotius* might have a osmoregulatory *salt-in* strategy, based on their acidic proteomes, the presence of typically *salt-in* transporters and by the absence of compatible solutes transporters or pathways for their biosynthesis which could suggest a *salt-out* strategy. We could find proton-pumping rhodopsins in all *Halonotius* genomes and sensory rhodopsins in some, all absorbing in the green spectrum.

Finally, this study has also permitted the emended description of the genus *Halonotius* and the identification of the isolates as two novel species of this genus, for which we propose the new names *Halonotius terrestris* sp. nov. and *Halonotius roseus* sp. nov., whose descriptions are given below.

### Description of *Halonotius terrestris* sp. nov.

*Halonotius terrestris* (ter.res’ tris. L. fem. adj. terrestris of or belonging to the earth, terrestrial).

Cells are Gram-stain-negative, motile, short rods, sometimes pleomorphic with 1.1 × 1.7 μm (Supplementary Figure 5). Colonies are circular, entire, red-pigmented with 0.2 mm in diameter on R2A25% medium after 10 days of incubation at 37°C. Extremely halophilic, able to grow in media with 15–30% (w/v) salts, with optimal growth at 25% (w/v) salts. No growth occurs in the absence of NaCl. Able to grow in the pH range of 7.0–7.5 and from 25 to 50°C, with optimal growth at pH 7.0 and at 37°C. Catalase and oxidase positive. Gelatin, starch, Tween 80 and esculin are not hydrolyzed. Nitrate and nitrite are reduced without gas production. H_2_S production is negative. Simmons’ citrate and Voges-Proskauer tests are negative. Methyl red test is positive. Indole is not produced. L-arginine, L-lysine and L-ornithine decarboxylase tests are negative. Acid is produced from D, L-arabinose, D-fructose, D-ribose and D-xylose but not from D-amygdalin, arbutin, D-cellobiose, L-citruline, dulcitol, D, L-ethionine, D-galactose, glycerol, D-glucose, inulin, lactose, D-maltose, D-mannitol, D-mannose, D-melezitose, D-melibiose, D-raffinose, sorbitol, D-sucrose, D-trehalose and L-xylitol. D-galactose, D-glucose, gycerol, maltose, D-mannitol, L-raffinose and tartrate are used as carbon and energy source but not D-arabinose, butanol, D-cellobiose, D-dulcitol, D-ethanol, fructose, lactose, D-mannose, D-melibiose, methanol, D-melezitose, D-ribose, salicin, sorbitol, sucrose, D-trehalose, xylitol and D-xylose. Not able to use L-alanine, L-cysteine, glutamine, L-methionine, L-glycine, L-lysine, isoleucine and valine as sole carbon, nitrogen and energy source but it uses L-arginine.

The major polar lipids are PG, PGP-Me, PGS and one glycolipid chromatographically identical to sulfated diglycosil diether (S-DGD-1). The DNA G + C content is 61.9 mol% (genome).

The type strain is F15B^T^ (= CECT 9688^T^ = CCM 8954^T^), isolated from a hypersaline soil of the Odiel Saltmarshes (Huelva, Spain).

The GenBank/EMBL/DDBJ accession number for the 16S rRNA and *rpoB*′ gene sequences of *Halonotius terrestris* F15B^T^ are MH465135 and MH454087, respectively, and that of the complete genome is RKLU00000000.

### Description of *Halonotius roseus* sp. nov.

*Halonotius roseus* (ro’se.us. L. masc. adj. roseus rose colored, pink).

Cells are Gram-stain-negative, non-motile, pleomorphic with 2.8 × 1.1 μm (Supplementary Figure 6). Colonies are circular, entire, red pigmented with 0.1 mm in diameter on R2A25% medium after 10 days of incubation at 37°C. Extremely halophilic, able to grow in media with 15–30% (w/v) salts, with optimal growth at 25% (w/v) salts. No growth occurs in the absence of NaCl. Able to grow in the pH range of 7.0–9.0 and from 25 to 50°C, with optimal growth at pH 7.5 and at 37°C. Catalase and oxidase positive. Gelatin, starch, Tween 80 and esculin are not hydrolyzed. Nitrate is reduced to nitrite, but nitrite is not reduced. Able to produce H_2_S. Simmons’ citrate, Voges-Proskauer and methyl red tests are negative. Indole is not produced. L-Arginine, L-lysine, and L-ornithine decarboxylase tests are negative. Acid is produced from D-ribose but not from D-amygdalin, D, L-arabinose, arbutin, D-cellobiose, L-citruline, dulcitol, D, L-ethionine D-fructose, D-galactose, glycerol, D-glucose, inulin, lactose, D-maltose, D-mannitol, D-mannose, D-melezitose, D-melibiose, D-raffinose, sorbitol, D-sucrose, D-trehalose, L-xylitol and D-xylose. Sucrose, butanol, tartrate, and D-xylose are used as carbon and energy source but not D-arabinose, D-cellobiose, D-dulcitol, D-ethanol, fructose, galactose, D-glucose, glycerol, lactose, maltose, D-mannitol, D-mannose, D-melezitose, D-melibiose, methanol, L-raffinose, D-ribose, salicin, sorbitol, D-trehalose and xylitol. Not able to use L-arginine, L-cysteine, glutamine, L-methionine, L-glycine, L-lysine, isoleucine and valine as sole carbon, nitrogen and energy source but it uses L-alanine.

The major polar lipids are PG, PGP-Me, PGS and one glycolipid chromatographically identical to sulfated diglycosil diether (S-DGD-1). The DNA G + C content is 62.7 mol% (genome).

The type strain is F9-27^T^ (= CECT 9745^T^ = CCM 8956^T^), isolated from the water of a pond from Isla Cristina saltern (Huelva, Spain).

The GenBank/EMBL/DDBJ accession number for the 16S rRNA and *rpoB’* gene sequences of *Halonotius roseus* F9-27^T^ are MK478811 and MK491894, respectively, and that of the complete genome is SESI00000000.

### Emended Description of the Genus *Halonotius* Burns et al. (2010)

Characteristics are as given in the description of the genus by Burns et al. (2010). The genus belongs phylogenetically to the family *Halorubraceae*, within the order *Haloferacales*, class *Halobacteria*. The major polar lipids are PG, PGP-Me, PGS and one glycolipid chromatographically identical to sulfated diglycosil diether (S-DGD-1). The DNA G + C content ranges from 59.5 to 62.7 mol% (genome).

## Data Availability

The datasets generated for this study can be found in GenBank, RKLU00000000 and SESI00000000.

## Author Contributions

AV, CS-P, and RG conceived and designed the study. AD-V, CS-P, and AV designed and performed the acquisition of environmental isolates. AD-V performed the microbial experiments and obtained the genomes. AD-V performed the bioinformatic analyses under close guidance of A-SA and RG. AD-V, A-SA, RG, CS-P, and AV analyzed and interpreted the data. AD-V, CS-P, and AV drafted the manuscript. AD-V, A-SA, RG, CS-P, and AV critically revised the manuscript. All authors read and approved the final manuscript.

## Conflict of Interest Statement

The authors declare that the research was conducted in the absence of any commercial or financial relationships that could be construed as a potential conflict of interest.

## Footnotes

1 https://doi.org/10.6084/m9.figshare.9125300.v1
