# Supplementary material for: New Halonotius Species Provide Genomics-Based Insights Into Cobalamin Synthesis in Haloarchaea
Source: Front Microbiol. 2019 Aug 27;10:1928. doi: 10.3389/fmicb.2019.01928 (PMC6719526; doi:10.3389/fmicb.2019.01928)

***Supplementary Material***

**New *Halonotius* species provide genomics-based insights into cobalamin synthesis in haloarchaea**

Ana Durán-Viseras^1^, Adrian-Stefan Andrei^2^, Rohit Ghai^2^, Cristina Sánchez-Porro^1^ and Antonio Ventosa^1^

^1^Department of Microbiology and Parasitology, Faculty of Pharmacy, University of Sevilla, 41012 Sevilla, Spain

^2^Department of Aquatic Microbial Ecology, Institute of Hydrobiology, Biology Centre of the Czech Academy of Sciences, České Budějovice, Czech Republic

**Supplementary Table 1.** Amino acid biosynthesis pathways found in *Halonotius* genomes.

| **Amino acid** | **Completeness** |
| --- | --- |
| Arginine | Complete |
| Cysteine | Complete |
| Histidine | Incomplete |
| Isoleucine | Complete |
| Leucine | Incomplete |
| Lysine | Complete |
| Ornitine | Complete |
| Phenylalanine | Incomplete |
| Proline | Complete |
| Serine | Incomplete |
| Threonine | Complete |
| Tryptophan | Complete |
| Tyrosine | Incomplete |
| Valine/Isoleucine | Complete |

**Supplementary Figure 1. Relations between genomic characteristics.**

Relations between genomic characteristics (i.e. estimated genome size, GC content, coding density, intergenic spacer length, and numbers of:coding DNA sequences, sigma factors, defense mechanisms and stop codon usage patterns) of *Halonotius* and selected prokaryotes that thrive in hypersaline environments. A linear regression is shown for the direct relationship between genome size and total coding DNA sequences.


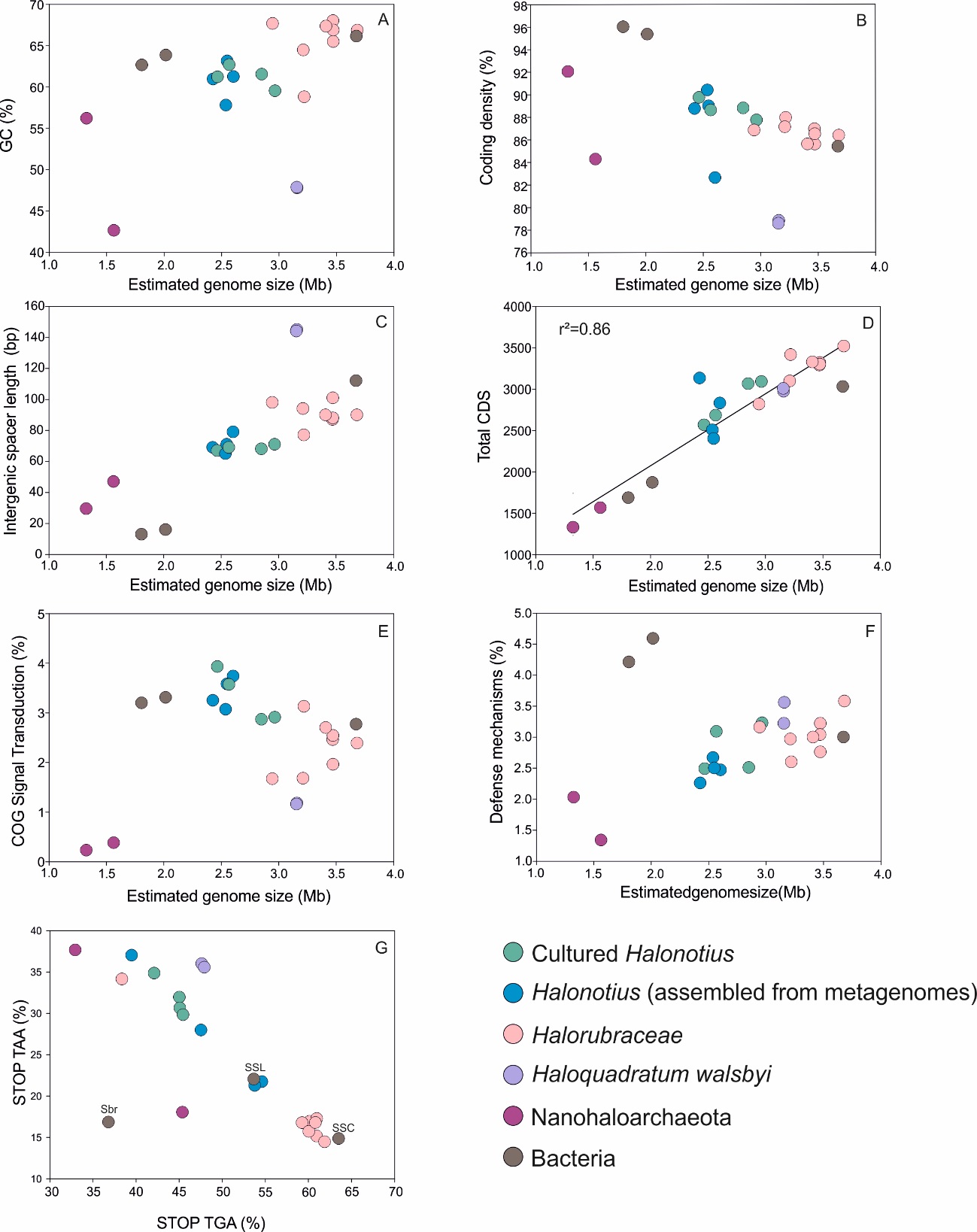


**Supplementary Figure 2. Recruitment plots of *Halonotius* strains against the metagenomic dataset SS37.**

A-D) Recruitment plots of *Halonotius* strains F13-13^T^, *Halonotius pteroides* CECT 7525^T^, F15B^T^ and F9-27^T^ and against the metagenomic dataset SS37. In each panel the Y axis represents the identity percentage and X axis represents the genome length. A restrictive cut-off 95 % of nucleotide identity in at least 30 bp of the metagenomic read was used. The black dashed line shows the threshold for presence of same species (95% identity).

**Abbreviations:** SS37 – Metagenome from Santa Pola solar saltern (Alicante, Spain)


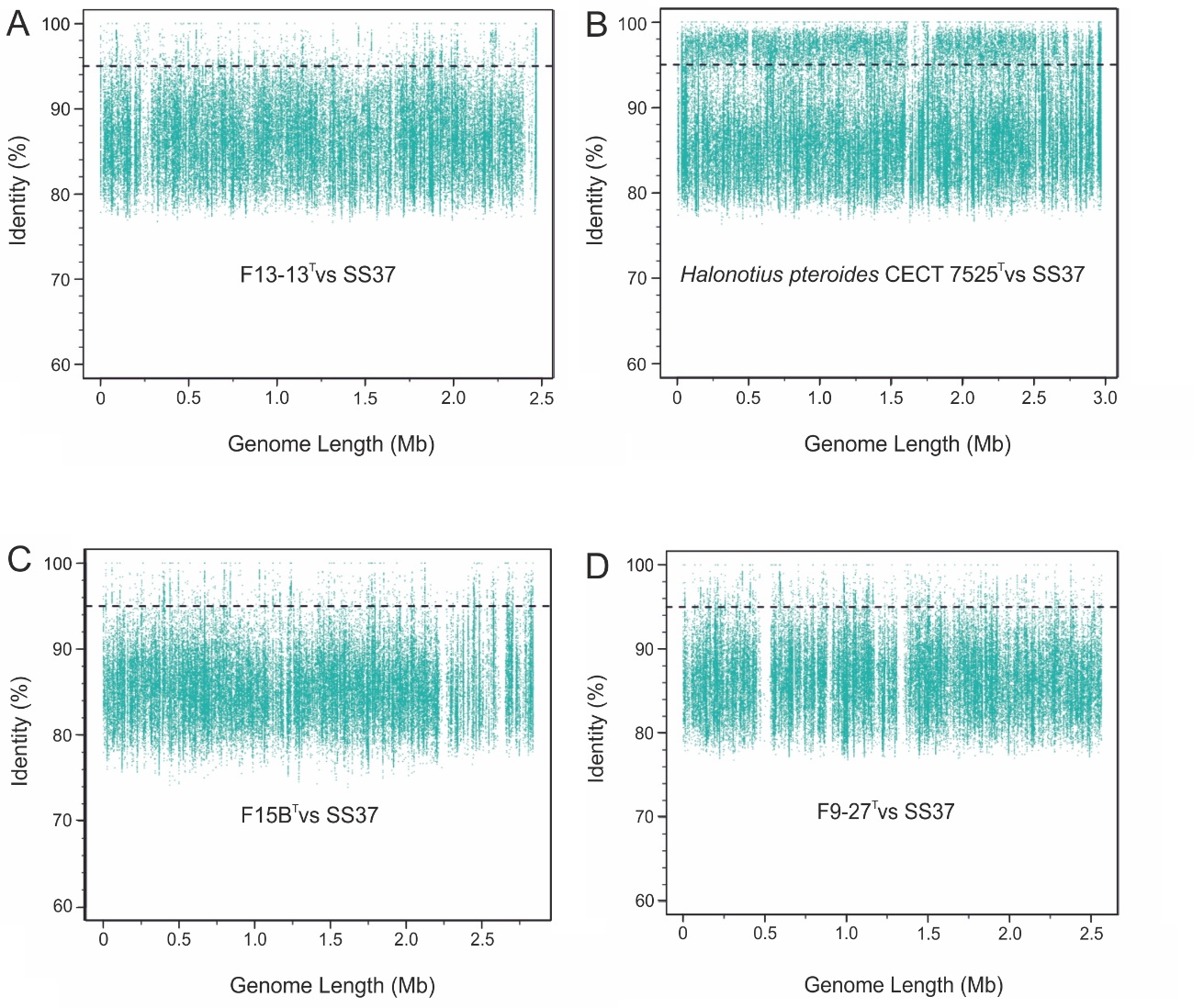


**Supplementary Figure 3. Worldwide distribution of the genus *Halonotius*.**

Global distribution of *Halonotius* 16S rRNA gene sequences. Colored circles are used to highlight the locations (as present in SILVA Database) where 16S rRNA gene sequences belonging to *Halonotius* were found. Arrow indicates the place where the strains (used in this study) were isolated. Black circumference in Australia indicates the place from were environmental *Halonotius* MAGs were recovered. The map is a modified version of a freely available map (<https://www.naturalearthdata.com/>).


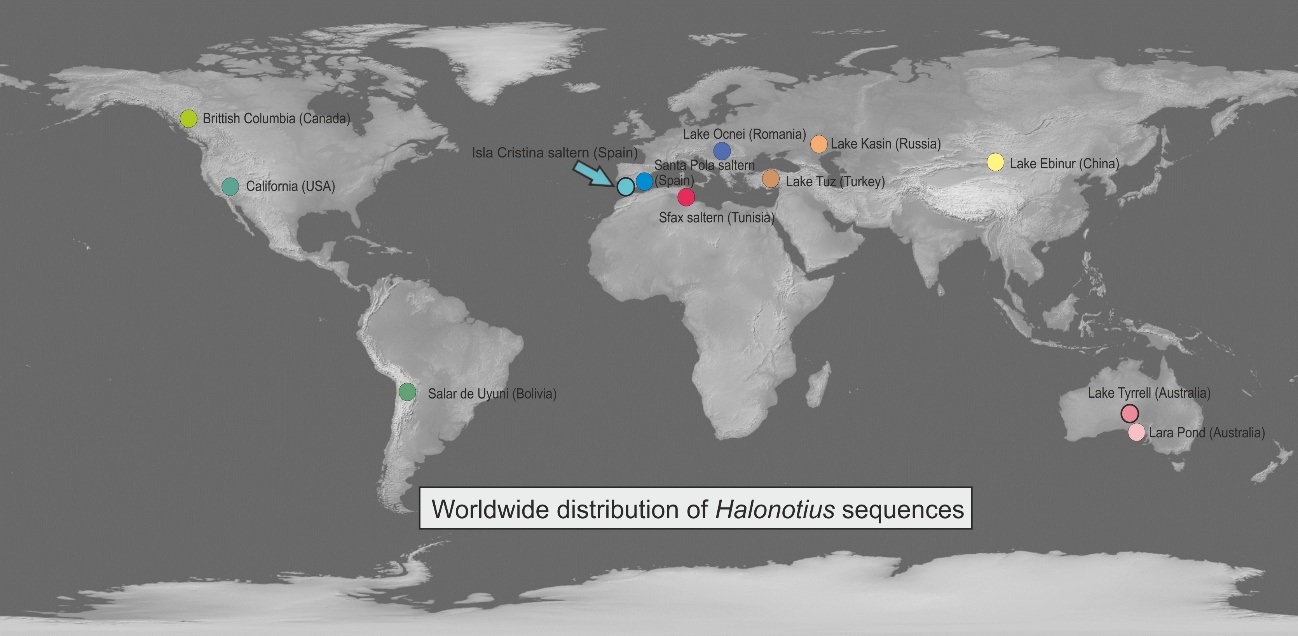


**Supplementary Figure 4. High performance thin layer chromatography (HPTLC) of *Halonotius* strains.**

**A)** Comparison of the polar lipids profile between *Halonotius* strains and some other haloarchaeal species. The plate was revealed with sulfuric acid 5 % in water, followed charred by heating at 160 ºC**. B)** Comparison of the phospholipids profile between *Halonotius* strains and some other haloarchaeal species. The plate was revealed with molybdenum blue spray reagent. **C)** Comparison of the total lipids profile between *Halonotius* strains and some other haloarchaeal species. The plate was revealed with fosfomolibdic acid. **Lanes:** 1, *Halobacterium salinarum* DSM 3754^T^; 2, *Halonotius aquaticus* F13-13^T^; 3, *Halonotius terrestris* sp. nov. F15B^T^; 4, *Halonotius roseus* sp. nov. F9-27^T^; 5*, Halonotius pteroides* CECT 7525^T^; 6, *Halohasta litorea* JCM 17270^T^; 7, *Halohasta litchfieldiae* JCM 15066^T^; 8, *Halorubrum saccharovorum* DSM 1137^T^

**Abbreviations:** BPG, biphosphatidylglycerol; PG, phosphatidylglycerol; PGP-Me, phosphatidylglycerol phosphate methyl ester; PGS, phosphatidylglycerol sulfate; S-DGD-1, sulphated diglycosil diether; S-TGD-1-PA, glycocardiolipin (sulfated triglycosyl diphytanyl archaeol ester linked to phosphatidic acid); S-TeGD, sulphated tetraglycosil diether.


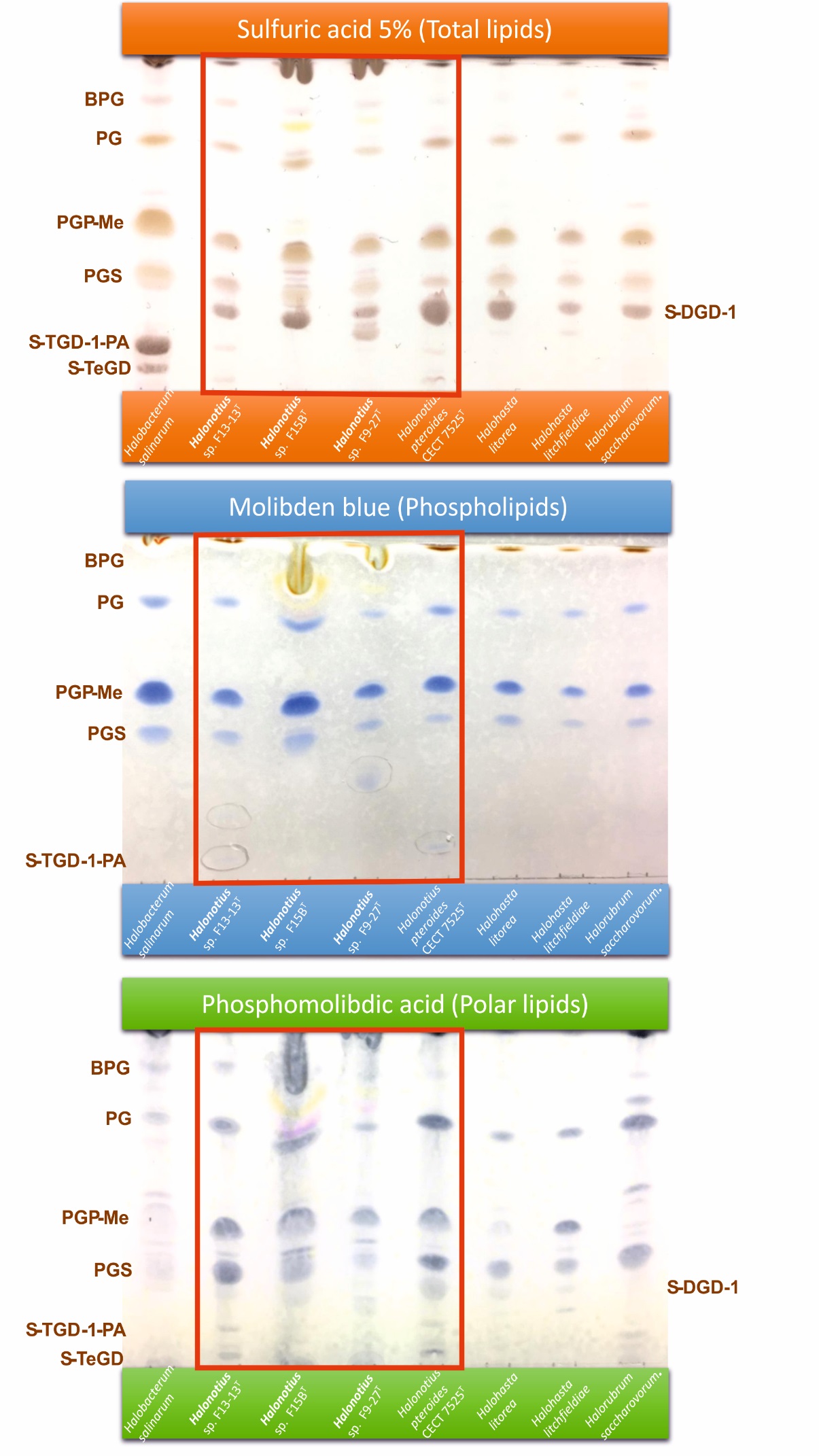


**Supplementary Figure 5. *Halonotius terrestris* sp. nov. F15B^T^ photomicrograph.**

Phase-contrast photomicrograph of cells of strain F15B^T^ cultured in liquid medium under optimal conditions. Scale bar, 10 μm.

**
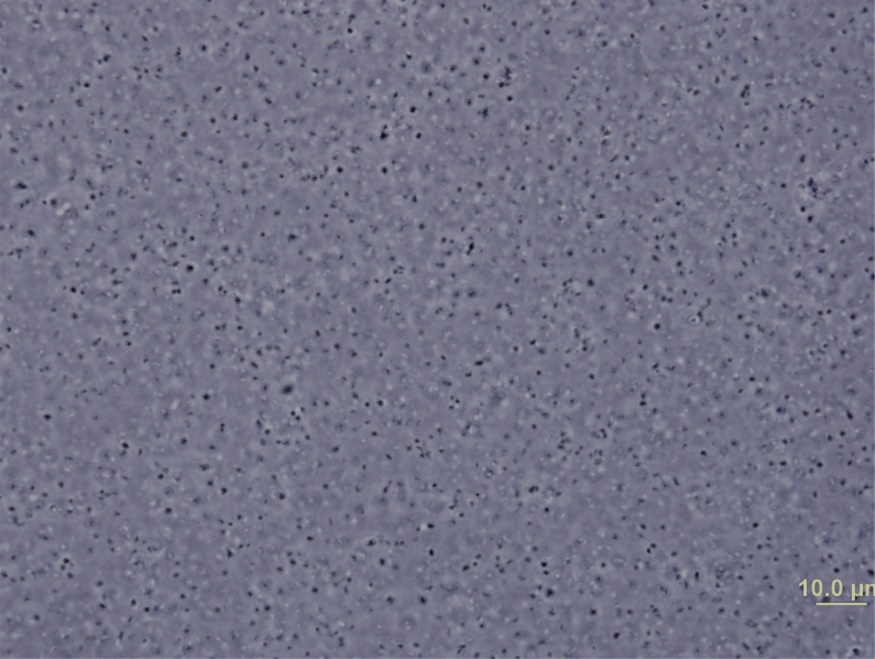
**

**Supplementary Figure 6. *Halonotius roseus* sp. nov. F9-27^T^ photomicrograph.**

Phase-contrast photomicrograph of cells of strain F9-27^T^ cultured in liquid medium under optimal conditions. Scale bar, 10 μm.


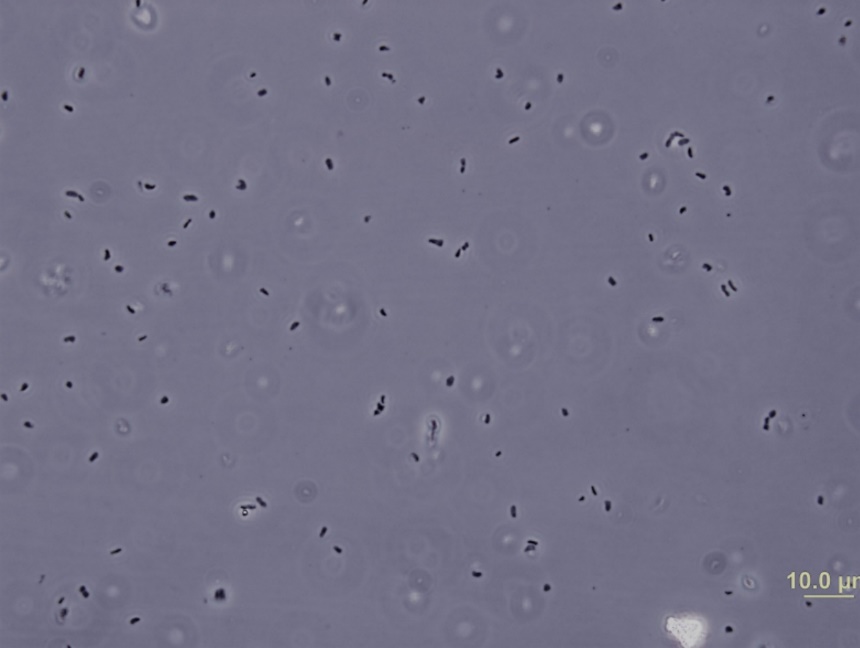

Supplement: Supplementary file 1 [file Data_Sheet_1.docx]
